# The association between meteorological variables and road traffic injuries: a study from Macao

**DOI:** 10.7717/peerj.6438

**Published:** 2019-02-12

**Authors:** Chon-Fu Lio, Hou-Hon Cheong, Chon-Hou Un, Iek-Long Lo, Shin-Yi Tsai

**Affiliations:** 1 Macau Association of Health Service Executives, Macau SAR, China; 2 Department of Laboratory Medicine, Mackay Memorial Hospital, Taipei, Department of Medicine, Graduate Institute of Long-Term Care, Graduate Institute of Biomedical Sciences, Mackay Medical College, New Taipei City, Taiwan; 3 Department of Health Policy and Management, Johns Hopkins University Bloomberg School of Public Health, Baltimore, MD, United States of America

**Keywords:** Road traffic injuries, Road safety, Meteorological factors, Road traffic accidents, Macao, Trauma, Injury, Weather

## Abstract

**Objective:**

Correlation analysis and multiple linear regression analysis were conducted to estimate the influence of meteorological factors on road traffic injuries stratified by severity. Crash rate was defined as mean monthly road traffic accidents per 1,000 vectors.

**Design:**

Ecological time-series study.

**Setting:**

Macao traffic accident registry database between January 1st, 2001 and November 31st, 2016.

**Participants:**

In total, 393,176 traffic accidents and 72,501 cases of road traffic injuries (RTIs) were enrolled; patients’ severity was divided into mild injury, required hospitalisation, and death.

**Exposure:**

Variation of monthly meteorological factors.

**Main outcome measure:**

Weather-condition-related road traffic accidents, injuries, and deaths.

**Results:**

Windy weather significantly correlated with increased number of traffic accidents among all transport vectors (*r* = .375 to .637; *p* < 0.001). Multiple linear regression showed temperature (*B* = 0.704; *p* < 0.05) and humidity (*B* =  − 0.537; *p* < 0.001) were independent factors for mild injury. The role of windy weather was relatively more obvious among patients with severe injuries (*B* = 0.304; *p* < 0.001) or those who died (*B* = 0.015; *p* < 0.001). A longer duration of sunshine was also associated to RTI-related deaths (*B* = 0.015; *p* < 0.001). In total, 13.4% of RTIs were attributable to meteorological factors and may be preventable.

**Conclusion:**

The World Health Organization stated that RTIs are a major but neglected public health challenge. This study demonstrates meteorological factors have significant effects on any degree of RTIs. The results may not be generalized to other climates or populations while the findings may have implications in both preventing injuries and to announce safety precautions regarding trauma and motor vehicle collisions to the general public by public agencies.

## Introduction

Road traffic injuries (RTIs) are becoming a major problem for public health systems worldwide due to their unpredictability (Gao et al., 2016; Zhang et al., 2015). According to the World Health Organization (WHO), RTIs were the ninth major cause of death globally in 2015 and the primary reason for death among people aged between 15 and 29 years (WHO, 2015a; Zhang et al., 2015). More than 1.2 million people have died and nearly 50 million people are injured annually worldwide (Gao et al., 2016; Rusli et al., 2017; Xie et al., 2016; Zhang et al., 2015; Zhang et al., 2011). Low- and middle-income countries account for approximately 90% of global RTIs (Alam & Mahal, 2016; Gao et al., 2016; Rusli et al., 2017; Xie et al., 2016; Zhang et al., 2015; Zhang et al., 2011). Without appropriate improvement in policies to prevent road traffic accidents (RTAs), RTIs are predicted to become the seventh leading cause of death worldwide by 2030, overtaking HIV/AIDS (Xie et al., 2016). In addition to the physical burden, RTIs can also lead to long-term psychological consequences (Mayou, Bryant & Duthie, 1993; Stallard, Velleman & Baldwin, 1998; Thompson, McArdle & Dunne, 1993). RTIs are also becoming an economic burden, with 3% of GDP lost globally and almost 5% of GDP lost in low- and middle-income countries each year (Xie et al., 2016). Therefore, the Decade of Action for Road Safety (DARS) 2011–2020 was established to reduce and stabilise the number of road traffic fatalities (Johal, Schemitsch & Bhandari, 2014; Xie et al., 2016).

In China, which has rapidly urbanised and is classified as a middle-income country by the WHO, the number of reported road traffic deaths is the highest in the world (National Bureau of Statistics of China, 2012; WHO, 2015a; Xie et al., 2016; Zhang et al., 2015). In 2013, 62,945 people died from RTIs, and the death rate per 100,000 people was 18.8 (Xie et al., 2016). In 2015, 187,781 RTAs were registered and 58,022 people died from RTIs. Furthermore, the direct property losses from RTIs exceeded 10 billion RMB (2015a). Although a decreasing trend in the number of RTAs has been evident since 2004, the property losses from RTIs remain high (Gao et al., 2016).

In light of the aforementioned statistics, the factors affecting the severity of RTIs and the occurrence of RTAs should be studied. The Haddon matrix model can be applied to determine the factors causing RTAs and consequently, to mitigate the severity of RTIs (National Bureau of Statistics of China, 2015; Masoumi et al., 2016; Runyan, 1998). According to the model, the factors affecting the occurrence and severity of RTIs comprise three main elements, namely human, vehicle, and environmental factors (Gao et al., 2016; Masoumi et al., 2016; Runyan, 1998). For example, road user behaviour is a very important factor that can cause RTAs, and improving road user behaviour is one goal of the DARS (Xie et al., 2016). Apart from the human and vehicle factors, meteorological factors, which are a type of environmental factor, are also critical; they account for approximately 20% of all RTA causes. Moreover, the contribution of meteorological factors to RTAs is unclear (Gao et al., 2016; Shankar et al., 2004). Thus, investigating and clarifying the relationship between meteorological factors and RTAs is essential to establish appropriate road traffic management.

Numerous studies have explored the relationship between weather conditions and RTIs with contradictive results due to different study designs, definitions of adverse weather, definition of trauma, localities and population groups, thus correlation with injury severity varies in existing literature. Therefore, this study investigated the relationship between meteorological factors and the severity of RTIs.

## Method

### Study design and data collection

This study consisted of a complete retrospective review of RTIs in the Macao Special Administrative Region that happened between January 1st, 2001 and November 31st, 2016. Macao is one of the autonomous territories in China, located on the western side of the Pearl River Delta. As of 2016, the population is approximately 650,000 and the land area is approximately 30.5 km^2^ without woodland; in short, Macao is a densely populated urban region. Although most meteorological studies related to RTIs obtained data in a medium-to-large city, the unevenly distributed meteorological conditions within the city may obscure the true correlation between RTIs and weather. By contrast, Macao’s unique geographic profile improved the reliability and applicability of our results.

All information related to traffic accidents and the severity of RTIs is mandatorily notified and recorded under the standardised operating procedures of the Public Security Police Force and all hospitals in Macao. Additionally, these data are verified and published monthly by the Statistics and Census Service of the Macao government (http://www.dsec.gov.mo/home_enus.aspx). This public database classifies the severity of RTIs into the following three categories: “mild injury” (injured victims who were sent to an emergency room by ambulance but did not require hospital admission), “required hospitalisation” (injured victims who were sent to an emergency room by ambulance and required hospital admission), and “death” (victims who did not survive before admission). Consequently, the collected mean monthly RTI cases from the government database are less likely to be affected by the limitation of a single hospital or emergency-based study, for which underestimation of the actual incidence rate of accidents with mild injury could be seen. Thus, the results of this study could reflect the actual situation of RTIs in Macao.

We recorded daily meteorological data from January 1st, 2001 to November 31st, 2016 by accessing the website of the Macao Meteorological and Geophysical Bureau (http://www.smg.gov.mo/smg/e_index.htm). We emphasised some meteorological parameters in this study, including daily barometric pressure (hPa), mean temperature (°C), diurnal amplitude (°C), dew temperature (°C), relative humidity (%), duration of sunshine (hours), wind speed (kn; 1 kn = 1,852/3,600 m/s), and rainfall (mm). Then, we calculated monthly averages of these factors and utilised them in the linear regression model.

### Statistical analysis

**Figure 1:**
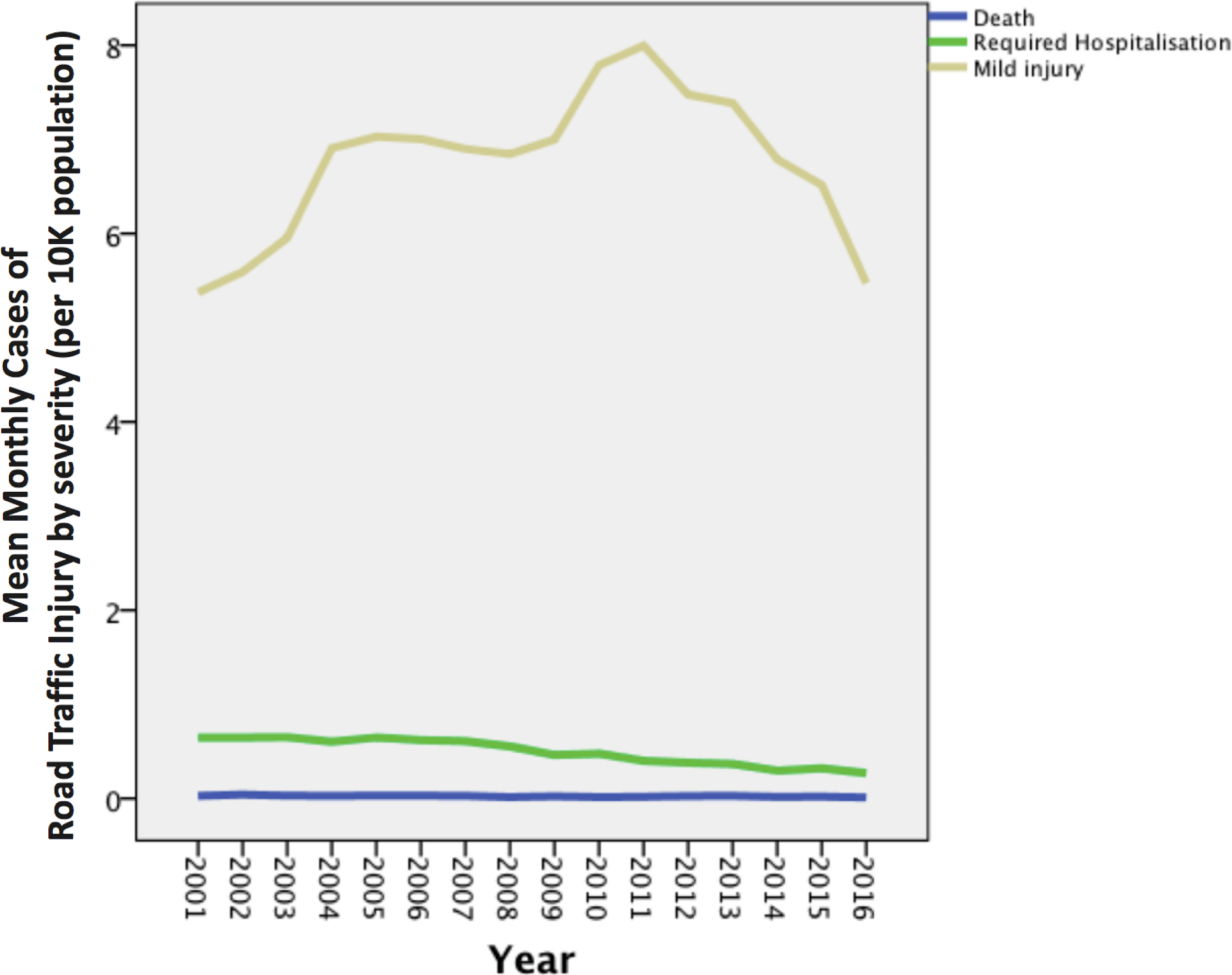
A time-series plots to display the distribution of the incidence rate of RTIs according to severity.

We used descriptive statistics in time-series plots to display the distribution of the incidence rate of RTIs according to severity (Fig. 1). Before the analysis, data exploration of all dependent variables was performed to determine the normality through a Shapiro–Wilk test. To establish adequate normality, we converted non-normally distributed variables through Templeton’s two-step transformation: first, percentile ranking was performed resulting in uniformly distributed probabilities; second, the inverse-normal transformation was applied yielding normally distributed *z*-scores (GF, 2011). Because fatal injuries are uncommon in Macao, we eliminated months without a death event to achieve greater normality for the linear regression model; therefore, only 130 months were included. Afterward, we conducted Pearson’s and Spearman’s correlation. Next, we entered the statistically significant parameters of RTIs into a stepwise multiple linear regression analysis (backward elimination) and verified the statistical assumptions and normality of the dependent variables in all regression models (Table 1). A comprehensive scatter plot shows all the correlation between predictors (Table S2). Finally, we tested the variance inflation factors (VIFs) to ensure a low level of multicollinearity in the model (all VIFs <5). In all of the comparisons, a *p* value of <0.05 signified statistical significance. We performed the statistical analyses using the SPSS software, Version 17.0 (SPSS Inc., Chicago, IL, USA).

**Table 1:** Normality tests of various types of outcome variables for linear regression analysis.

**Monthly incidence of**	**Mean**	**Std. Deviation**	**Skewness**	**Kurtosis**	***P*value(Shapiro–Wilk)**
Mild injury	68.61	10.59	0.12	−0.65	0.17
Injury required hospitalisation	5.13	0.15	0.26	−0.13	0.63
Fatal injury^a^	0.36	0.02	0.04	−0.41	0.58

**Notes.**

a We eliminated months without a death event to achieve greater normality for the linear regression model; 130 months were included.

## Results

After a thorough exploration of the public database from the Statistics and Census Service, we enrolled 191 consecutive months of data related to meteorology and RTIs in our dataset. Table 2 presents a summary of the characteristics of meteorological conditions. Table S1 shows the pattern of meteorological factors versus time. In total, we enrolled 72,501 cases of RTIs. To determine the correlation between meteorological factors and RTI outcomes, we divided the cases into three categories of injury severity, namely “mild (injury” (*n* = 67, 461, 93.0%), “required hospitalisation” *n* = 4798, 6.6%), and “death” (*n* = 242, 0.3%). This dataset, obtained from government statistical reports, enabled us to estimate the true prevalence of mild injuries, which do not usually require admission and are not registered in hospital databases; this minimised the potential bias caused by undercounting mild RTIs in our study. The large sample size and long study period in a super-urbanised, small city was ideal for the injury–meteorology analysis. Mild RTIs were positively correlated with mean temperature and duration of sunshine, but negatively correlated with barometric pressure and relative humidity (all *p* values < 0.05) in Table 3. We further investigated the significant meteorological variables through multiple linear regression models. The results (Table 4) revealed that mean temperature (*B* = 0.704; 95% CI [0.250–1.159], *p* < 0.05) and relative humidity (*B* =  − 0.537; 95% CI [−0.823 to −0.252], *p* < 0.001) were independent factors affecting mild RTIs (adjusted *R*^2^ = 8.8%). Notably, RTIs requiring hospitalisation were only correlated with wind speed (*B* = 0.304; 95% CI [0.228–0.380], *p* < 0.001) in the multivariate model (adjusted *R*^2^ = 24.5%). Finally, duration of sunshine (*p* < 0.001) and wind speed (*p* < 0.001) contributed to 9.7% of the fatal RTIs. It is worth noting that some constant term is non-significant which means that predictive value may become unreliable when considering the case of all the meteorological factors are zero. More detailed modeling information could be referred to Tables S3–S5.

**Table 2:** Descriptive statistics of meteorological variables from 2001 to 2016 in Macao.

	**Mean**	**Median**	**Std. Deviation**	**Minimum**	**Maximum**
Barometric pressure (hPa)	1,013.09	1,014.00	5.748	1,002	1,023
Mean temperature (C°)	22.68	23.84	5.00	11.53	29.17
Diurnal amplitude (C°)	5.47	5.46	0.82	3.76	7.84
Dew temperature (C°)	18.63	19.60	5.56	5.78	26.01
Relative humidity (%)	79.07	80.97	7.36	57.52	94.53
Duration of sunshine (hours)	4.77	4.89	1.90	0.56	10.22
Wind speed (Knots)	13.51	13.90	2.85	7.33	19.45
Rainfall (mm)	5.20	2.96	5.84	0.00	40.13

**Table 3:** Correlations between meteorological variables and the monthly cases of road traffic injuries per 100,000 people stratified by injury severity.

	**Mild injury**	**Required hospitalization**	**Death**
Barometric pressure (hPa)	r_p_ = − .147^*^	r_p_ = − .046	r_p_ = − .023
Mean temperature (C°)	r_p_ = .149^*^	r_p_ = .096	r_p_ = .095
Diurnal amplitude (C°)	r_p_ = .048	r_p_ = − .044	r_p_ = .101
Dew temperature (C°)	r_p_ = .058	r_p_ = .055	r_p_ = .043
Relative humidity (%)	r_p_ = − .223^**^	r_p_ = − .106	r_p_ = − .159
Duration of sunshine (hours)	r_s_ = .184^*^	r_s_ = .063	r_s_ = .187^*^
Wind speed (knots)	r_s_ = − .117	r_s_ = .538^**^	r_s_ = .203^*^
Rainfall (mm)	r_p_ = .085	r_p_ = .051	r_p_ = − .057

**Notes.**

r_p_ refers to Pearson’s correlation coefficient, and r_s_ refers to Spearman’s rank correlation coefficient.

** Correlation is significant at the 0.01 level (2-tailed).

* Correlation is significant at the 0.05 level (2-tailed).

**Table 4:** Multivariate analysis to determine the influence of meteorological factors on the monthly case of road traffic injuries per 100,000 people stratified by injury severity in Macau: Linear regression models.

Meteorological factors	**Mild injury**^a^		**Required hospitalization**^b^		**Death**^c^	
****	**B**	**95% CI**	**B**	**95% CI**	**B**	**95% CI**
Constant	98.778	(77.609, 119.946)^*^	0.865	(−0.186, 1.915)	0.063	(−0.117, 0.242)
Mean temperature (C° )	0.704	(0.250, 1.159)^*^	–	–	–	–
Relative humidity (%)	−0.537	(−0.823, −0.252)^**^	–	–	–	–
Duration of sunshine (hours)	−0.996	(−2.268, 0.337)	–	–	0.019	(0.002, 0.036)^*^
Wind speed (Knots)	–	–	0.304	(0.228, 0.380)^**^	0.015	(0.004, 0.026)^*^

**Notes.**

* indicates *p* < 0.05.

** indicates *p* < 0.001.

a *F* = 7.127 (*p* < 0.001); adjusted *R*^2^ = 0.088; barometric pressure was eliminated in this model due to multicollinearity (VIF >5) between mean temperatures; maximal Cook’s distance = 0.040; root mean squared error = 93.991.

b *F* = 62.287 (*p* < 0.001); adjusted *R*^2^ = 0.245; maximal Cook’s distance = 0.048; root mean squared error = 2.307.

c *F* = 5.083 (*p* < 0.001); adjusted *R*^2^ = 0.069; maximal Cook’s distance = 0.063; root mean squared error = .031.

## Discussion

Following the rapid development of the tourism and gaming industries, the number of motor vehicles in Macao has increased by approximately 5.2% per year, from 115,770 in 2001 to 249,339 in 2015 (Macao SAR, 2015; Stallard, Velleman & Baldwin, 1998). It could possibly imply that rapid up surging needs of trucks might be a core factor in contributing a relatively increased case of RTIs during the years. This phenomenon may also be due to an increase of exposure (e.g., number of km driven).

Although the yearly incidence rate of RTI-related deaths remained steady and that of RTIs that required hospitalisation declined gradually from 2001 to 2016, the yearly incidence rate of mild RTIs was several times higher than the more serious patients, with an upward trend from 2001 to 2011 (Fig. 1). Legislation of the traffic law “Lei do Trânsito Rodoviário, number 3/2007” was administered in 2007, in which drunk driving was criminalised in the law, together with heavier penalties towards other traffic related regulations. It could possibly explain the decline in RTIs-required hospitalisation.

Macao is a peninsula surrounded by the sea on three sides; it is exposed to direct solar radiation twice a year with a subtropical oceanic monsoon climate that is characterised by intense radiation, exuberant evaporation, adequate moisture, high temperature, and abundant rainfall. The area is in a typical monsoon climate zone because of the clear circulation conversion between winter and summer, resulting in relatively short spring and autumn seasonal interchange periods. Consequently, the impact of a heat wave cannot be overemphasised. Numerous studies have shown that high temperatures lead to increased accident frequency, (Scott, 1986) which may be due to increased traffic intensity (Cools, Moons & Wets, 2010), and increased outdoor activities putting people at risk of traumatic injuries and motor vehicle collisions (Abe et al., 2008). Our data showed the effect of 1 °C of additional mean monthly temperature increases the number of mild injuries by 0.7 per 100,000 population. In accordance with previous research, the results of this study suggest that temperature is positively associated with RTIs, particularly with mild injuries. Bergel-Hayat et al. claimed that 1 °C of additional average monthly temperature increases the number of injury accidents in that month by 1%–2% (Bergel-Hayat et al., 2013).

Satterthwatte et al. reported that the glare of sunlight can also make the driving more difficult (Satterthwaite, 1976), and Nofal & Saeed, (1997) revealed that most accidents occur during the noon rush hour of 12–3 pm, when sunlight is most intense. A recent report similarly showed that an increased number of RTIs was associated with additional sunshine hours (Gao et al., 2016). The report also indicated that the core contributing factor was the average increase in traffic activity and intensity associated with extensive sunshine, which may lead to a negative effect on road safety (Al Hassan & Barker, 1999; Mario, Elke & Geert, 2010). In the current study, a longer duration of sunshine was found to be correlated with fatal RTIs.

Numerous studies had investigated the impact of rainfall intensity towards traffic volume and RTI, but results were contradictory. Most of the study suggested precipitation is associated with increased number of crashes (Caliendo, Guida & Parisi, 2007; Chang & Chen, 2005; Edwards, 1996; Smith, 1982), some showed negative association (Christoforou, Cohen & Karlaftis, 2010; Theofilatos, Graham & Yannis, 2012), and the other revealed no association (Haghighi-Talab, 1973; Sherretz & Farhar, 1978) even after considering the injury severity (Gao et al., 2016). Intuitively, rainfall is thought to increase the number of RTIs due to the reasons such as decreased friction between the road surface in contact with tyres of vehicles, increased difficulty of vehicle handling and restricted visibility. The explanation why there is non-significant correlation between rainfall and RTIs in this study may be due to drivers’ compensation to bad weather, decreased traffic flow, and the adaptation of the local population to the regular rainy time of year known as plum rain season (Gao et al., 2016).

Available evidences suggested no straightforward correlation between relative humidity and RTIs (Gao et al., 2016; Theofilatos & Yannis, 2014). One study suggested that rainfall is positively associated with RTIs (Rusli et al., 2017; Theofilatos & Yannis, 2014). Conversely, our data revealed a significant negative correlation between relative humidity and monthly mild RTIs. One possible hypothesis maybe proposed regarding the association between humidity and precipitation, as well as slippery road conditions which raise driver’s awareness and decrease traffic volume leading a decreased accident rate.

Current studies regarding the effect of wind speed on RTIs are inconsistent. One recent report observed a negative association between wind speed and RTIs through correlation analysis and a regression model (Gao et al., 2016). However, most studies have suggested that wind speed has a positive relationship with RTIs, because high wind speed can eventually lead to increased difficulty in vehicle control and thereby increases the number of crashes (Hermans et al., 2006; Qiu & Nixon, 2008). In the present study, we found that wind speed played an important role in the monthly RTIs that required hospitalisation and those that lead to death. However, our study revealed no statistically significant associations between RTIs and other meteorological factors such as diurnal amplitude, rainfall, and dew temperature.

A previous study showed that an approximate 20% of traffic accidents could be attributed to meteorological factors. Complementarily, this study further analysed the association with specified degree of injury severity. Our results indicated that 13.4% of RTIs can be explained by meteorological factors, of which weather conditions had the greatest influence on required hospitalization RTIs (*R*^2^ = 24.5%); however, in mildly injured patients and those who were deceased, the importance of these factors was less obvious, with only 8.8% and 6.9% of such accidents ascribed to meteorological factors, respectively. We argue that this lower-than-expected influence is reasonable because many other factors, including the mechanisms of injury, type of transportation, safety protections provided by individual manufacturers, underlying diseases, and emergency medical resource response time, can influence the prognosis of traumatic injury. For example, although the number of accidents has not significantly decreased, the fatality rate is slowly declining in high-income countries (WHO, 2015b). Rapid progress in medical development promotes survival in seriously injured patients. Moreover, other factors, such as changes in regulations and policies, development of advanced pavement architecture, and automotive safety systems, play crucial roles in reducing the poor outcomes after traffic accidents (Ernstberger et al., 2015).

This study had the following strengths and values. First of all, studies have investigated the relationships between interactive host–agent–environment factors and the occurrence and severity of RTIs through the concepts of primary, secondary, and tertiary prevention after William Haddon introduced the Haddon matrix model in 1968. However, controversial results were noted on the associations between meteorological factors and RTIs due to different models derived from studies with different designs, definitions of trauma, localities and population groups, and this study provides results stratified according to injury and vehicle type. Second, obtaining all the collected data, including monthly RTIs and meteorological data, from the Macao Special Administrative Region government database, provided legitimacy and credibility and reduced the possibility of misclassification. Third, this is the first study in Macao focusing mainly on RTIs and meteorological factors, comprising a 191-month study period from 2001 to 2016 and 72,501 RTI cases. We contend that our results are sufficiently statistically robust to reveal various correlations. Moreover, our study included patients with mild injuries, which we found to be associated with higher mean temperature and relative humidity; Previous researches have largely ignored these participants because of limited access to their data in hospital-based studies where trauma admission rates and fatality were mainly focused. In fact, a study indicated only 22.5% of cases resulted in admission to hospital (Abe et al., 2008).

Our study also had some limitations. First, despite the inclusion of a relatively large number of RTIs, the number of RTI-related deaths was small compared with the large number of mild injuries and RTIs requiring hospitalisation. Thus, the relationship between meteorological factors and mortality was unclear in the current study. Second, the weather data and associations in our study only represent a region with a subtropical climate. We were unable to investigate associations between low temperature conditions (such as freezing temperatures, snow and fog), and RTIs because of the climatic restriction of Macao. Although rainfall is a common weather parameter that was proved to be associated with increased number of RTIs, our study echoed Gao et al. results with no significant positive association shown. It could possibly be the inappropriateness of applying the measurement of rainfall (mm) as the parameter marker instead of investigating the association of RTIs in hourly-based or in a real-time model approach. Third, we did not consider other potential factors, such as the effects of holidays, weekdays, and the number of tourists (tourism is a crucial industry in Macao). Besides, other weather parameter such as air pollution (e.g., PM 2.5) was not included in current study (Guo et al., 2018). Moreover, although we made an initial classification of the injury severity, this only expressed an approximation of RTI severity that it differs from those applied in previous studies, such as the KABCO system; detailed information to stratify the minor injuries in the database is unavailable. Hence, more comprehensive demographic and detail traumatic injury grading data are needed for future studies. Indeed, the contributions of meteorological factors to the occurrence of RTIs could be modified and affected by other human, vehicle, and environmental factors such as vehicle speed, traffic flow and density, traffic volume, pedestrian behaviour, and number of passenger kilometres. Additionally, local factors such as traffic legislation, newly emerging public transport companies, pavement construction, lack of highways, and the rapid expansion residential and working populations are potential confounding factors for the occurrence of RTIs. We did not include these modifying factors or adjust for them as covariates in our analysis because of the difficulties with obtaining, possessing, and quantifying the relevant data. However, our results are consistent with those of other studies in which our regression model has a similar explanatory power. Finally, we calculated monthly intervals in our regression models because daily data was unavailable in Macao. Therefore, bias may be introduced because monthly intervals are prone to oversimplification and thus are less suitable for measuring weather influences. Moreover, excessive stratification may undermine the normality of the data because of the dilution of the number of casualties in each day, reducing the reliability and validity of the regression model. Future study may try Autoregressive Integrated Moving Average model to gain a better prediction value via controlling the lagging of variables, and long-term/seasonal trend.

## Conclusion

This study demonstrates that 13.4% of RTIs are attributable to meteorological factors. Meteorological factors played an important role in any severity of RTIs, particularly the effect of wind speed on the numbers of severe injury and death cases. The results may not be generalized to other climates or populations while the findings may have implications for controlling traffic flow and advising travellers during adverse weather.

##  Supplemental Information

10.7717/peerj.6438/supp-1 Table S1 The pattern of meteorological factors during the calibrating period All the parameters seem stable and periodic over calibration period except the variation of wind speed becomes greater in recent years.

10.7717/peerj.6438/supp-2 Table S2 Comprehensive scatter plots to show all the correlation between predictors

10.7717/peerj.6438/supp-3 Table S3 Stepwise multiple linear regression analysis (backward elimination) for the associations between monthly mild injury cases related to road traffic injury and meteorological factors

10.7717/peerj.6438/supp-4 Table S4 Stepwise multiple linear regression analysis (backward elimination) for the associations between monthly hospitalisation cases related to road traffic injury and meteorological factors

10.7717/peerj.6438/supp-5 Table S5 Stepwise multiple linear regression analysis (backward elimination) for the associations between monthly death cases related to road traffic injury and meteorological factors

10.7717/peerj.6438/supp-6 Supplemental Information 6 Raw data table

## Additional Information and Declarations

### Data Availability

The following information was supplied regarding data availability: The raw data are available in a Supplemental File.
