# Supplementary material for: The association between meteorological variables and road traffic injuries: a study from Macao
Source: PeerJ. 2019 Feb 12;7:e6438. doi: 10.7717/peerj.6438 (PMC6376939; doi:10.7717/peerj.6438)
Supplement: Table S1 — All the parameters seem stable and periodic over calibration period except the variation of wind speed becomes greater in recent years. [file peerj-07-6438-s001.docx]

| 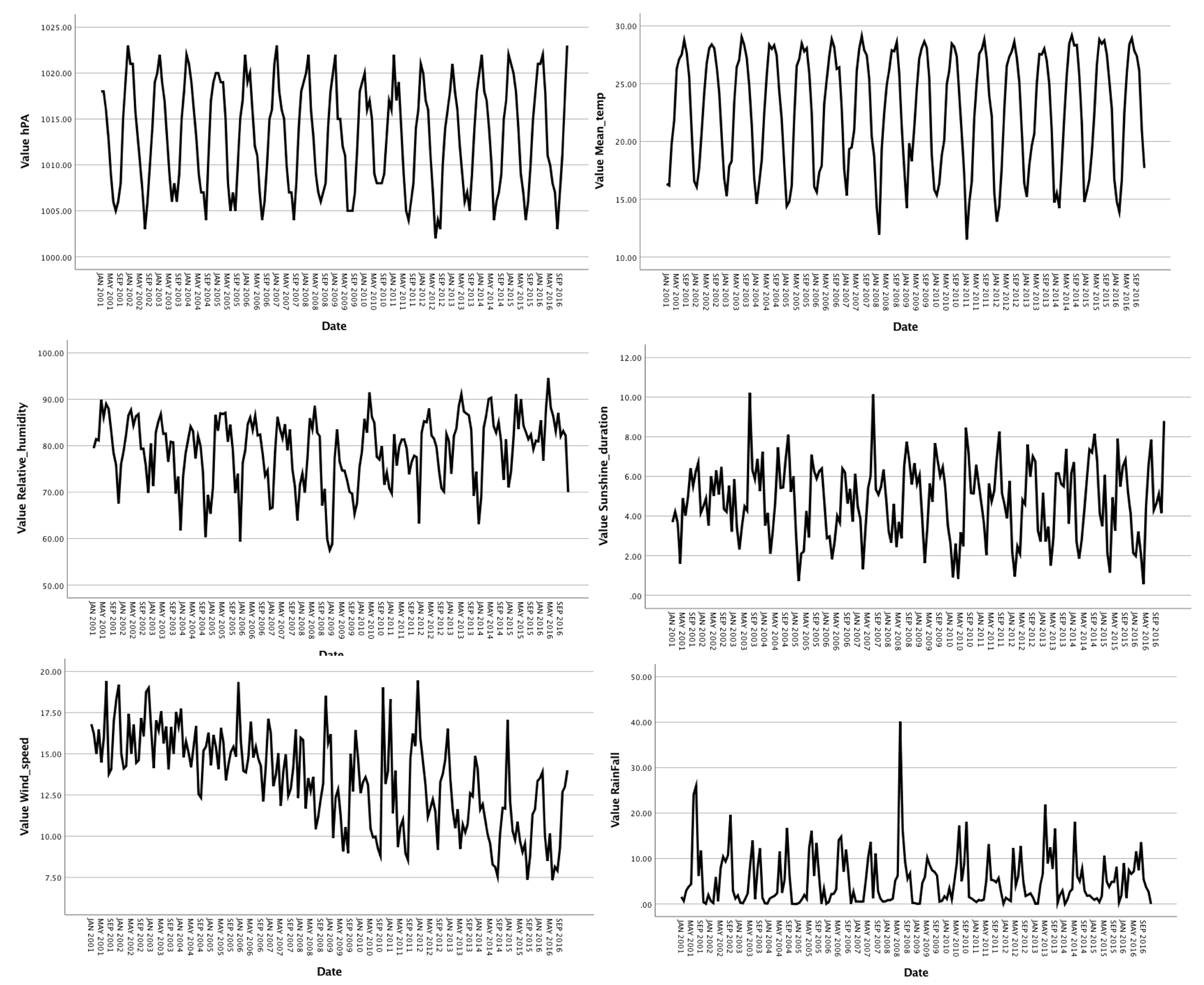 |
| --- |
| **Table S1. The pattern of meteorological factors during the calibrating period.**  All the parameters seem stable and periodic over calibration period except the variation of wind speed becomes greater in recent years. |
