# Supplementary figures and images for: The association between meteorological variables and road traffic injuries: a study from Macao
Source: PeerJ. 2019 Feb 12;7:e6438. doi: 10.7717/peerj.6438 (PMC6376939; doi:10.7717/peerj.6438)

| 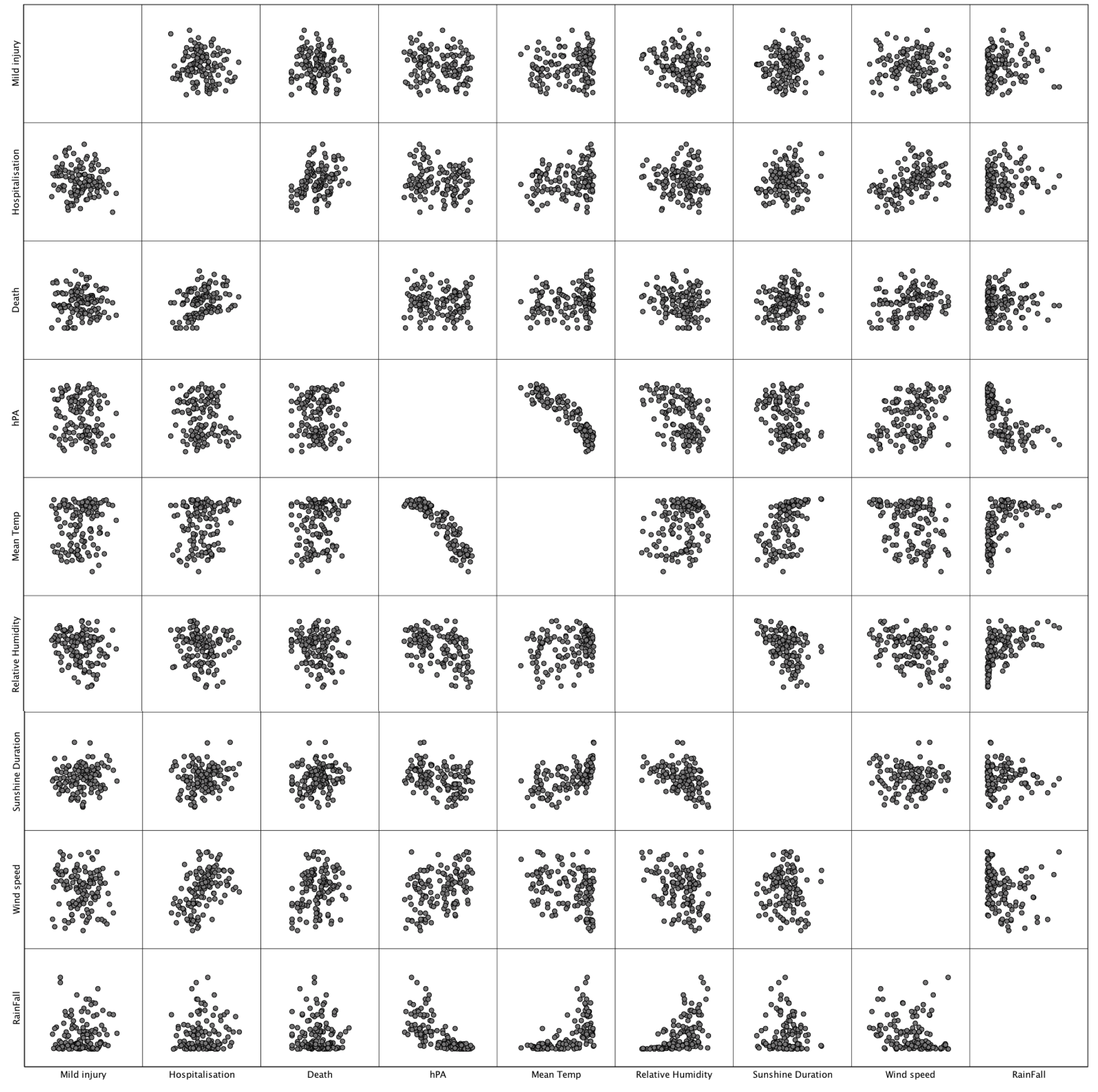 |
| --- |
| **Table S2. Comprehensive scatter plots to show all the correlation between predictors** |

Supplement: Table S2 [file peerj-07-6438-s002.docx]
