# Supplementary material for: The association between meteorological variables and road traffic injuries: a study from Macao
Source: PeerJ. 2019 Feb 12;7:e6438. doi: 10.7717/peerj.6438 (PMC6376939; doi:10.7717/peerj.6438)
Supplement: Table S3 [file peerj-07-6438-s003.docx]

| **Table S3. Stepwise multiple linear regression analysis (backward elimination) for the associations between monthly mild injury cases related to road traffic injury and meteorological factors.** | | | | | | | | | |
| --- | --- | --- | --- | --- | --- | --- | --- | --- | --- |
|  | **Variables** | **Standardized Coefficients** | | | **95%CI of β** | **Collinearity Statistics** | **ANOVA Analysis** | | **Adjusted R Square** |
|  |  | **β** | **t** | **Sig** |  | **VIF** | **F** | **Sig** |  |
| **Model 1** | Constant | 98.778 | 9.206 | <.001 | (77.609, 119.956) |  | 7.229 | <.001 | .090 |
|  | Mean temperature (C^o^) | .704 | 3.058 | .003 | (.250, 1.159) | 2.667 |  |  |  |
|  | Relative humidity (%) | -.537 | -3.711 | <.001 | (-.823, -.252) | 2.285 |  |  |  |
|  | Duration of sunshine (hours) | -.966 | -1.463 | .145 | (-2.268, .337) | 3.118 |  |  |  |
| **Model 2** | Constant | 87.882 | 7.748 | <.001 | (72.597, 103.167) |  | 9.714 | <.001 | .084 |
|  | Mean temperature (C^o^) | .443 | 3.033 | .003 | (.155, .732) | 1.067 |  |  |  |
|  | Relative humidity (%) | -.383 | -3.856 | <.001 | (-.578, -.187) | 1.067 |  |  |  |
