# Supplementary material for: The association between meteorological variables and road traffic injuries: a study from Macao
Source: PeerJ. 2019 Feb 12;7:e6438. doi: 10.7717/peerj.6438 (PMC6376939; doi:10.7717/peerj.6438)
Supplement: Table S4 [file peerj-07-6438-s004.docx]

| **Table S4. Stepwise multiple linear regression analysis (backward elimination) for the associations between monthly hospitalisation cases related to road traffic injury and meteorological factors.** | | | | | | | | | |
| --- | --- | --- | --- | --- | --- | --- | --- | --- | --- |
|  | **Variables** | **Standardized Coefficients** | | | **95%CI of β** | **Collinearity Statistics** | **ANOVA Analysis** | | **Adjusted R Square** |
|  |  | **β** | **t** | **Sig** |  | **VIF** | **F** | **Sig** |  |
| **Model 1** | Constant | .865 | 1.623 | .106 | (-.186, 1.915) |  | 62.287 | <.001 | .245 |
|  | Wind speed (Knots) | .304 | 7.892 | <.001 | (.228, .380) | 1.000 |  |  |  |
