# Supplementary material for: The association between meteorological variables and road traffic injuries: a study from Macao
Source: PeerJ. 2019 Feb 12;7:e6438. doi: 10.7717/peerj.6438 (PMC6376939; doi:10.7717/peerj.6438)
Supplement: Table S5 [file peerj-07-6438-s005.docx]

| **Table S5. Stepwise multiple linear regression analysis (backward elimination) for the associations between monthly death cases related to road traffic injury and meteorological factors.** | | | | | | | | | |
| --- | --- | --- | --- | --- | --- | --- | --- | --- | --- |
|  | **Variables** | **Standardized Coefficients** | | | **95%CI of β** | **Collinearity Statistics** | **ANOVA Analysis** | | **Adjusted R Square** |
|  |  | **β** | **t** | **Sig** |  | **VIF** | **F** | **Sig** |  |
| **Model 1** | Constant | .063 | .691 | .491 | (-.117, .242) |  | 5.752 | .004 | .069 |
|  | Duration of sunshine (hours) | .019 | 2.173 | .032 | (.002, .036) | 1.003 |  |  |  |
|  | Wind speed (Knots) | .015 | 2.723 | .007 | (.004, .026) | 1.003 |  |  |  |
